# A Simple Secret Key Generation by Using a Combination of Pre-Processing Method with a Multilevel Quantization

**DOI:** 10.3390/e21020192

**Published:** 2019-02-18

**Authors:** Mike Yuliana, Wirawan, Suwadi

**Affiliations:** 1 Department of Electrical Engineering, Faculty of Electrical Technology, Institut Teknologi Sepuluh Nopember, Jalan Raya ITS, Keputih, Sukolilo, Surabaya 60111, Indonesia; 2 Department of Electrical Engineering, Politeknik Elektronika Negeri Surabaya (PENS), Jalan Raya ITS, Keputih, Sukolilo, Surabaya 60111, Indonesia

**Keywords:** secret key generation, modified Kalman, combined multilevel quantization

## Abstract

Limitations of the computational and energy capabilities of IoT devices provide new challenges in securing communication between devices. Physical layer security (PHYSEC) is one of the solutions that can be used to solve the communication security challenges. In this paper, we conducted an investigation on PHYSEC which utilizes channel reciprocity in generating a secret key, commonly known as secret key generation (SKG) schemes. Our research focused on the efforts to get a simple SKG scheme by eliminating the information reconciliation stage so as to reduce the high computational and communication cost. We exploited the pre-processing method by proposing a modified Kalman (MK) and performing a combination of the method with a multilevel quantization, i.e., combined multilevel quantization (CMQ). Our approach produces a simple SKG scheme for its significant increase in reciprocity so that an identical secret key between two legitimate users can be obtained without going through the information reconciliation stage.

## 1. Introduction

The Internet of Things (IoT) is one of the results of advancing network and telecommunications technology. This technology is expected to connect millions of home devices, vehicles, and industrial environments. Some applications that can be developed from this IoT device include autonomous vehicles [1], health services [2], industrial services [3], and smart homes [4]. It is conceivable that millions of devices will be equipped with various sensors and be connected to the internet through various heterogeneous networks. IoT, therefore, can be said as a system that allows the transfer of data between interconnected devices without human intervention. IoT infrastructure is connected to a communication network to collect and exchange information between devices. Referring to the basic character of wireless media, data aggregation through wireless communication is very vulnerable to eavesdropping [5,6,7]. This condition is a serious threat to the security of the IoT network, thus demanding the need for research dedicated to the security of wireless communication in collecting/exchanging data [6] and providing a system for hardware security [8].

The conventional symmetrical cryptographic protocol requires the distribution of secret key or certificate management to ensure the security of the data being transmitted [9,10,11,12], where the security of this protocol is influenced by the computing capability of the device. With the development of computing capabilities of the eavesdropping device (for example by using quantum computing technology), however, the protocol will be easily solved in the future [13,14,15]. Besides, the distribution of a secret key is also very complex to implement on a large scale network because it requires intensive secret key distribution to support the establishment of secret keys between devices. Several studies focus on efforts to obtain cheap and promising solutions from symmetric cryptography and can be used as a lightweight cryptographic solution on IoT devices [16,17,18,19,20,21]. The SKG scheme is one solution that is often used for several reasons, i.e., it utilizing randomness from wireless channels so it is secure in theory, it can be conducted between a pair of users without the need for third-party assistance, and it is lightweight so it is suitable for IoT devices [5].

There are 3 properties of wireless channels that are often used in the SKG scheme, where the properties include reciprocity, spatial decorrelation, and they are spatial-temporal [22]. Reciprocity indicates that the wireless channel is symmetric, where the channels of legitimate users will be the same [23,24,25]. Spatial decorrelation can be fulfilled if the distribution is uniformly distributed and there are variations in channels due to scatterer movements, and also sending and receiving nodes. Eavesdroppers that are more than half the wavelength will get uncorrelated channel parameters with the legitimate users. Dynamic environmental conditions will also result in various wave propagation consequences such as scattering, diffraction, and reflection so the spatial-temporal character can be fulfilled. With the fulfillment of the spatial-temporal then the character of the channel parameters obtained are also expected to fulfill the randomness requirements. Besides environmental conditions, other factors that also affect the randomness of channel parameters are the coherence time of the channel.

Several types of channel parameters can be obtained from the communication between wireless devices, i.e., received signal strength (RSS) [26,27,28,29], channel impulse response (CIR) [30], and ultra-wideband (UWB) [31]. These parameters are obtained from the average signal strength provided by the physical layer within a certain time period. CIR and CSI parameters are difficult to explore in more detail since most wireless devices are not designed to display all channel information, so some studies have modified wireless card drivers. RSS parameters have their advantages, where the parameters can be obtained from most wireless devices without the need for modification. This is the basis for selecting RSS as a parameter to be extracted as the secret key in the implementation of the SKG scheme that will be built.

Generally, there are four stages used to generate a secret key, i.e., probing channel, quantization, information reconciliation, and privacy amplification [32,33]. The disadvantage of using these four SKG stages is the high mismatch bits that are produced between two users [34,35]. One of the solutions to solve this problem is the utilization of the pre-processing method before the quantization stage. This method performs smoothing or fitting, so it increases reciprocity between the two users and reduces the incompatibility of the bits produced. Some studies in [35,36,37,38,39] show that the utilization of this method can reduce the mismatch of bits produced but still requires an information reconciliation stage to correct the mismatch bits. This condition results in the increasing computational time needed, because of the increasing number of stages that must be passed. In addition, the information reconciliation stage also requires the exchange of parity bit between the two users, increasing the communication cost of the implementation of IoT device communication in real life.

In this paper, we focus on getting a simple SKG scheme by reducing the reconciliation stage, so it can reduce the high computational and communication cost of implementation. This paper has three main contributions. Firstly, a new pre-process method called modified Kalman (MK) is proposed. It combines the polynomial regression method with the modified Kalman filter. The results show that our approach can significantly increase reciprocity, so a high correlation can be obtained. Secondly, we combined MK method with a multilevel quantization called the combined multilevel quantization (CMQ) method. This method produces a simple SKG scheme for its significant increase in reciprocity, so it can obtain an identical secret key between two legitimate users without going through the information reconciliation stage. The stage reduction also resulted in less computational and communication cost. Thirdly, performance evaluation of our proposed SKG scheme was performed by using 802.11 devices in line of sight (LOS) and non-line of sight (NLOS) indoor environments.

The rest of this paper is organized as the following. Section 2 describes the system model used in the SKG scheme. Section 3 explains the detail of each stage from our proposed SKG scheme. Section 4 explains the experimental setup. In Section 5, the performance evaluation of the experiment is discussed. We conclude the paper in Section 6.

## 2. System Model

This section explains the modeling system used, and the principle of reciprocity as the main basis of the SKG scheme being built.

### 2.1. System Modelling on the Secret Key Generation (SKG) Scheme

**Figure 1:**
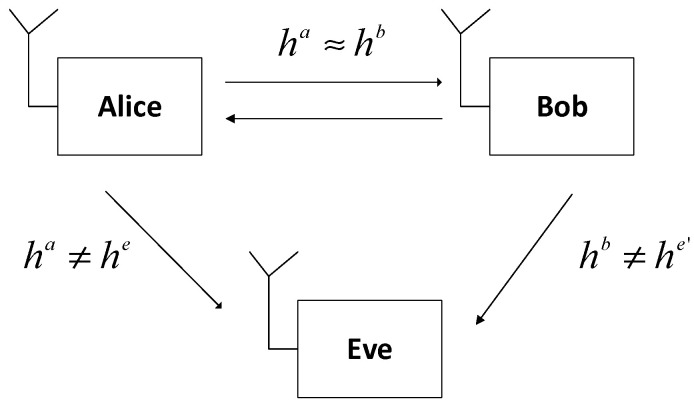
System modeling on the secret key generation (SKG) scheme.

Illustrations of the SKG scheme modeling can be seen in Figure 1. Two legitimate users, Alice and Bob, will generate wireless channel parameters $\left(h^{a},h^{b}\right)$. In this paper, we use RSS as a channel parameter because most IoT devices have the ability to do RSS measurements. Eve, who acts as an eavesdropper, attempts to intercept the communication carried out by Alice and Bob $\left(h^{e},h^{e^{\prime }}\right)$, where Eve is a passive attacker model. Each user works in half-duplex mode. Alice and Bob will get high correlated channel parameters if the measurement is conducted in coherence time, so the principle of channel reciprocity can be fulfilled. Meanwhile, Eve, which is more than half the wavelength, will not get a correlated parameter channel with the legitimate user. In our SKG scheme, we assume that all the procedures and parameters used by the legitimate user are known by Eve.

### 2.2. Channel Reciprocity

**Figure 2:**
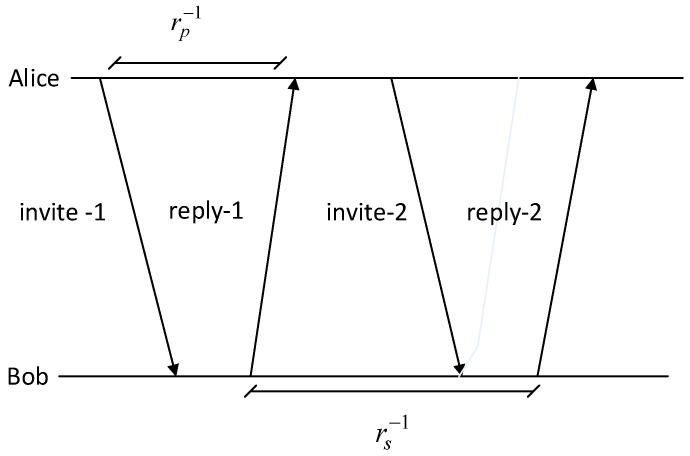
Measurement parameters.

Channel reciprocity is symmetrical characteristic of wireless channel parameters that allow for the formation of a secret key as an encryption key between two legitimate users. Wireless channel parameters are obtained by conducting measurements on each user, which is highly influenced by the characteristics of the various phenomena that characterize the wireless channel. Due to the limitations of the device which resulted in half duplex measurements, the SKG scheme sends and receives wireless channel parameters alternately. Two important parameters used in the measurement are the probing rate $r_{p}$ and sampling rate $r_{s}$ as shown in Figure 2. The principle of reciprocity can be fulfilled if the measurement of wireless channel parameters is carried out within coherence time $T_{c}$ where the channel is assumed to be fixed, while randomness requirements can be fulfilled if $r_{s}^{-1}>T_{c}$ [40]. Coherence time invite/reply is influenced by various physical phenomena and changes from time to time and space. For example, we use 802.11b standard devices that work on a 2.4 GHz carrier frequency $f_{c}$. In dynamic scenarios where user movements occur, variations in wireless channel parameters are influenced by the Doppler effect. If the user speed v is 1.2 m/s, then the Doppler frequency obtained is $f_{D}=vf_{c}/c=1.2x2.4x10^{9}/(3.10^{8})=9.6$ Hz. Coherence time obtained is $T_{c}=1/\left|f_{D}\right|=1/9.6=104.16$ ms. In our proposed SKG scheme, we use a sampling rate $r_{s}^{-1}$ of 110 ms. The sampling rate value has exceeded coherence time, so the randomness requirements have been fulfilled. Meanwhile, the probing rate is within coherence time, so the principle of channel reciprocity between two users also can be fulfilled.

## 3. Secret Key Generation Scheme

There are 3 parts described in this section, wherein the section includes existing SKG schemes, our proposed SKG schemes, and performance parameters.

### 3.1. Existing SKG Schemes

The procedure for obtaining a secret key from several previous studies generally consists of four stages. Firstly, by sending probe signals alternately between Alice and Bob to get wireless channel parameters called the probing stage channels. We use RSS channel parameters for ease of implementation, although there are some other channel parameters which have been discussed in several previous studies. Secondly, wireless channel parameters obtained from probing channel stage will be converted into the form of bits to obtain the initial key (quantization stage). Thirdly, information reconciliation stage to correct the mismatched bit in the initial key caused by noise and half duplex mode of the wireless device used. At this stage, the parity bit is exchanged to equalize the initial key, so a synchronized key can be obtained. The exchange of parity bit results in the leakage of the secret key making it easier for Eve to conclude the secret key generated. Fourthly, privacy amplification stage to increase the randomness of the secret key and verify the secret key generated.

The problem arising from the existence of the information reconciliation stage is that the increase in the cost of this stage is greatly influenced by the number of bit mismatches and the success of parity bit exchange. The more bit mismatch, the longer is the time needed to make corrections. The success of parity bit exchange is also strongly influenced by network conditions. If the network condition is poor, the longer is the time needed to exchange parity bit. In addition, parity bit exchanges also trigger the leakage of parity bit information to eavesdroppers, making it easier for eavesdroppers to get the same key. In this paper, we tried to solve these problems by proposing the pre-processing stage before quantization, so it can increase the reciprocity of RSS measurement results significantly. The combination of our proposed pre-process method with multilevel quantization is also able to reduce bit mismatches, so the identical secret keys can be obtained without requiring an information reconciliation stage.

### 3.2. Proposed SKG Schemes

**Figure 3:**
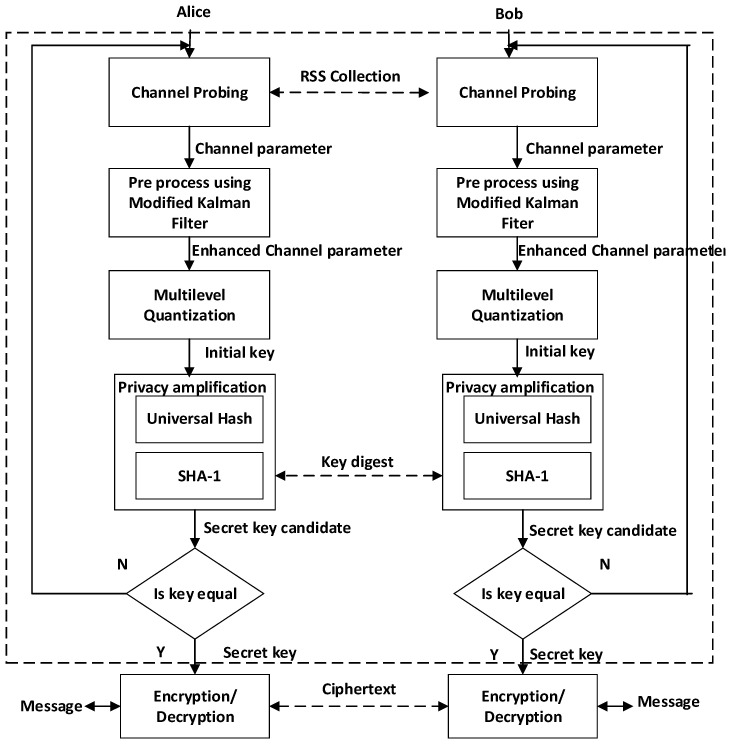
Our proposed SKG scheme.

Our proposed SKG scheme is shown in Figure 3 with the following four stages, i.e., channel probing, pre-process, multilevel quantization, and privacy amplification. To simplify the secret key generation procedure Alice was set as an initiator. Instead of directly changing the RSS measurement results into bits, we chose to add the pre-process stage using the MK method. Several studies [35,38] expose that the addition of the pre-processing method before the quantization will reduce the incompatibility of the resulting bits. Our proposed pre-process method i.e., modified Kalman is a combination of the polynomial regression and modified Kalman filter. At this stage, the RSS measurement results divide into several data blocks and our proposed method is used to increase the reciprocity of each block of data. The advantage of this mechanism is the pre-processing method, which is able to work more effectively so there is a significant increase in reciprocity for several blocks of data. This increase is indicated by an increase in the value of the correlation coefficient close to 1. The next stage is a combination of the MK method with the CMQ. Quantization is used to change the RSS pre-process results into bits. We use multilevel quantization to avoid too few bits being generated. At this stage, the quantization process is carried out on each pre-process data block. Several blocks of data from the pre-process have a very high similarity with a correlation coefficient close to 1. Thus, this increases the probability of acquisition of an identical secret key between two legitimate users without going through the information reconciliation stage. Compared to other studies that also add a pre-processing stage [35,36,37,38], we can show that our proposed SKG scheme is simpler because it can reduce the information reconciliation stage. The advantage of losing this stage is the reduced difficulty of implementation because there is no exchange of parity bits. Another advantage is the increased security of the SKG scheme that was built because it reduces the possibility of leaking information to the eavesdropper. In the privacy amplification stage, we add a universal hash [41] to increase the randomness of the generated key so as to meet the requirements of randomness and SHA-1 [42] in order to guarantee that the generated key between two legitimate users is the same.

In the probing channel stage, Alice and Bob measure the RSS channel parameters $h^{u}$, where the super-script u is replaced by a for Alice and b for Bob. However, because the wireless device used has a half-duplex mode, the two users cannot send and receive RSS simultaneously. We used the ping command to ensure that the invite and reply times from the channel parameter measurements do not exceed coherence time. At the completion of the channel probing stage, Two legitimate users will create sets of n RSS channel parameters which are expressed by Equations (1) and (2).

(1) $$h^{a}=\{h^{a}(t_{1}),h^{a}(t_{2}),\ldots ,h^{a}(t_{n})\}$$

(2) $$h^{b}=\{h^{b}(t_{1}^{\prime }),h^{b}(t_{2}^{\prime }),\ldots ,h^{b}(t_{n}^{\prime })$$

where $h^{a}$ is the RSS channel parameter measured by Alice and $h^{b}$ is the RSS channel parameter measured by Bob. In its implementation, although $h^{a}$ is not exactly the same as $h^{b}$ because of non-simultaneous measurements and noise, they will have a high correlation if bi-directional probing (for example $t_{1}^{\prime }-t_{1}$) is smaller than coherence time. During coherence time the channel is assumed to be stable, so $h^{a}\approx h^{b}$. Channel parameters received by the eavesdropper are defined by Equations (3) and (4):

(3) $$h^{e}=\{h^{e}(t_{1}^{\prime \prime }),h^{e}(t_{2}^{\prime \prime }),\ldots ,h^{e}(t_{n}^{\prime \prime })$$

(4) $$h^{e\prime }=\{h^{e\prime }(t_{1}^{\prime \prime \prime }),h^{e\prime }(t_{2}^{\prime \prime \prime }),\ldots ,h^{e\prime }(t_{n}^{\prime \prime \prime })$$

where $h^{e}$ is channel parameter measured by Eve from Alice, while $h^{e\prime }$ is channel parameter measured by Eve from Bob. It was assumed that Eve was more than ½ the wavelength from two legitimate users so it was difficult for Eve to generate the same key because $h^{e}$ and $h^{e\prime }$ did not correlate with $h^{a}$ and $h^{b}$.

The next stage is the pre-processed method which aims to increase the reciprocity of RSS channel parameters as measured by the two users. Our proposed pre-process method is the MK that combines the adopted polynomial regression (Algorithm 1) and modified the Kalman filter (Algorithm 2). In Algorithm 1, the RSS data measurement results $h^{u}$ are then divided into several blocks N to reach a number of blocks $N_{B}$ written as Equation (5).

(5) $$h_{N}=[h_{N}^{T}\ldots h_{N-N_{B}+1}^{T}]$$

Each $h_{N}$ contains a number of RSS data $S_{B}$. In this paper, we use the 2nd order polynomial regression, known as the quadratic for each RSS data block as shown in Equation (6).

(6) $$h_{\ell }=a_{0}+a_{1}x_{\ell }+a_{2}x_{\ell }{}^{2}+e$$

where $h_{\ell }$ is the RSS data at a time $x_{\ell }$ with $\ell =(1,2,\ldots ,S_{B})$, while e is RSS data discrepancy between Alice and Bob. The sum of the square of the residual $S_{r}$ is expressed by Equation (7).

(7) $$S_{r}=\sum_{\ell =1}^{S_{B}}(h_{\ell }-a_{0}-a_{1}x_{\ell }-a_{2}x_{\ell }^{2}{)}^{2}$$

The derivative of the equation is indicated by Equation (8).

(8) $$\frac{\partial S_{r}}{\partial a_{0}}=-2\sum (h_{\ell }-a_{0}-a_{1}x_{\ell }-a_{2}x_{\ell }^{2})\;\frac{\partial S_{r}}{\partial a_{1}}=-2\sum x_{\ell }(h_{\ell }-a_{0}-a_{1}x_{\ell }-a_{2}x_{\ell }^{2})\;\frac{\partial S_{r}}{\partial a_{1}}=-2\sum x_{\ell }^{2}(h_{\ell }-a_{0}-a_{1}x_{\ell }-a_{2}x_{\ell }^{2})$$

If the derivative of the Polynomial equation is made equal to zero then the normal equation is obtained as shown in Equation (9).

(9) $$(S_{B})a_{0}+(\sum x_{\ell })a_{1}+(\sum x_{\ell }^{2})a_{2}=\sum h_{\ell }\;(\sum x_{\ell })a_{0}+(\sum x_{\ell }^{2})a_{1}+(\sum x_{\ell }^{3})a_{2}=\sum x_{\ell }h_{\ell }\;\left(\sum x_{\ell }^{2}\right)a_{0}+(\sum x_{\ell }^{3})a_{1}+(\sum x_{\ell }^{4})a_{2}=\sum x_{\ell }^{2}h_{\ell }$$

where $a_{0},a_{1}$ and $a_{2}$ are 3 unknown Polynomial coefficient. We explain the detailed mechanism of the adopted polynomial algorithm that is run in each user in Algorithm 1. The way to calculate the element of normal equations in the form of an augmented matrix is shown on Lines 1–15 (Equation (9)), and the way for solving the augmented matrix to get 3 unknown polynomial coefficients by using elimination methods is shown on Lines 16–17. Finally, the output of pre-processing results by using adopted polynomial regression is shown on Line 20.
**Algorithm 1**: Adopted Polynomial Regression.**Input:** Channel measurement $h^{u}$ at the time $x_{\ell }$.**Input:** Block Number $N_{B}$, the size of each block $S_{B}$.**Input:** Polynomial coefficient a, order polynomial m.**Output:** Enhanced Polynomial Regression $y^{u}$
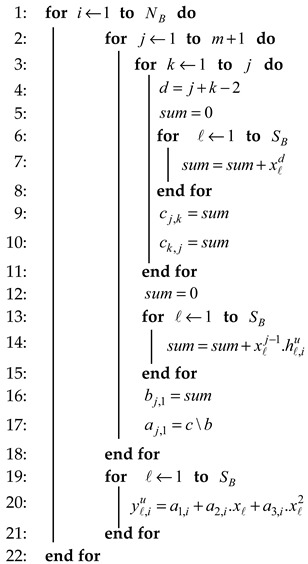


The MK method (Algorithm 2) works recursively to estimate status by using a priori and a posteriori estimation, where the estimation is carried out for each data block (the same as Algorithm 1). The initial estimation is carried out by the time update equation, while the correction of the estimation is carried out with the measurement update equation. The input of the time update equation is a priori estimation ${\hat{x}}_{k-1}$, a priori covariance error $P_{k-1}$, and covariance noise process Q. The equation of the time update is shown in Equation (10).

(10) $${\hat{x}}_{k}^{-}=A.{\hat{x}}_{k-1}\;P_{k}^{-}=A.P_{k-1}.A+Q$$

where A is a status of measurement at a time k−1. There are five inputs of the measurement update equation, i.e., ${\hat{x}}_{k}^{-}$, $P_{k}^{-}$, $y_{k}^{u}$, measurement noise covariance R and the variance of each data block v. We modified the correction process in the measurement update as shown in Equation (11).

(11) $$K_{k}=(P_{k}^{-}/(P_{k}^{-}+R)\;P_{k}=P_{k}^{-}(1-K_{k})\;{\hat{x}}_{k}={\hat{x}}_{k}^{-}+K_{k}(y_{k}^{u}-H{\hat{x}}_{k}^{-})\;z_{k}={\hat{x}}_{k}\;z_{k}=z_{k}+0.2v$$

where H is a status of measurement at a time k, ${\hat{x}}_{k}$ is a posteriori estimation, $P_{k}$ is a posteriori covariance error, $K_{k}$ is Kalman gain, while $z_{k}$ is RSS data as a result of the pre-processing stage with the MK method. We modified the a posteriori estimation results by adding 0.2 times the variance of each block of data and eliminate the parameter H with the aim of improving the reciprocity of pre-processing data $z_{k}$. The detailed mechanism of the MK method is explained in Algorithm 2. We initialize several parameters that provide the best configuration and shown on Lines 1–2. The time update process can be seen on Lines 4–5 and Lines 12–13 (Equation (10)), while the modified measurement update process can be seen on Lines 6–10 and Lines 14–18 (Equation (11)).

After the pre-processing stage, enhanced modified Kalman $z^{u}$ will be converted into bit by using multilevel quantization, so the initial key can be obtained. Like the previous stage, in this stage, RSS data will also be divided into several data blocks. Algorithm 3 shows our proposed method, i.e., CMQ, wherein this algorithm combines the MK method with multilevel quantization. Inputs from this algorithm are $z^{u}$, mean of each block $\mu^{u}$, and variance of each block $v^{u}$. The determination of the initial key bit is based on the Gray Code $b_{k}$ at each level, and the levels’ determinants are mean and variance for each block. Data outside the levels will be discarded. We select RSS amount of data per block to be quantized $S_{B}=128$ because it produces the best performance parameters when compared to the other number. The significance of reciprocity improvement from the pre-process stage results in several identical initial keys, so it does not require the information reconciliation stage. This stage requires synchronization in the communication and requires a good network connection. If the network connection is poor, the synchronization process will be repeated, resulting in the system being built inefficiently.
**Algorithm 2**: Modified Kalman (MK).**Input:** Enhanced Polynomial Regression $y^{u}$**Input:** Status of measurement at a time k−1
*A*, status of measurement at the time *k*
*H***Input:** co-variance of process noise *Q*, co-variance of measurement noise *R***Input:** A priori estimation ${\hat{x}}_{k}^{-}$, a priori covariance error $P_{k}^{-}$**Input:** A posteriori estimation ${\hat{x}}_{k}$, a posteriori covariance error $P_{k}$**Input:** Initial guesses ${\hat{x}}_{0}$, $P_{0}$**Input:** Block number $N_{B}$, the size of each block $S_{B}$, the variance for each block $v^{u}$**Output:** Enhanced modified Kalman $z^{u}$
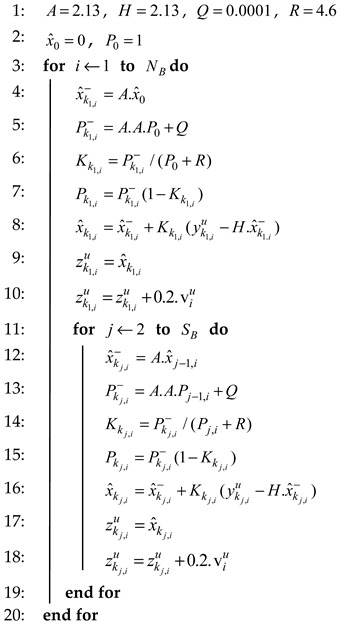


The initial key obtained as output from the quantization stage does not necessarily fulfill the randomness requirements. In the privacy amplification stage, we add a universal hash [41] to ensure a small number of crashes in expectancy by using specific arithmetical properties. The addition of this function will increase the randomness of the initial key so that it is expected to be able to fulfill the minimum approximate entropy requirements. To test the randomness of the initial key we conducted randomization testing using the National Institute of Standards and Technology (NIST) statistical suite. The initial key that fulfills the requirements will be processed further so that it can be used as a key to the cryptosystem. We use SHA-1 [42] to guarantee that the initial key generated by two legitimate users is the same. Alice as the initiator sends a key digest from several initial keys to Bob. Bob also generates a key digest and compares the results with Alice. The same key digest shows the same initial key bit. The same block of initial keys will be used as a candidate secret key, while different blocks will be discarded. SHA-1 produces a 160-bit message digest, so sending the entire message will result in high communication costs. In this paper, we only sent 6 bits from each block to obtain 98% correctness during the verification process [43].
**Algorithm 3**: Combined Multilevel Quantization (CMQ).**Input:** Enhanced channel parameter $z^{u}$**Input:** Block Number $N_{B}$, the size of each block $S_{B}$**Input:** The variance for each block $v^{u}$, means of each block ${µ}^{u}$, quantization level *Q***Output:** Initial key $K^{u}$
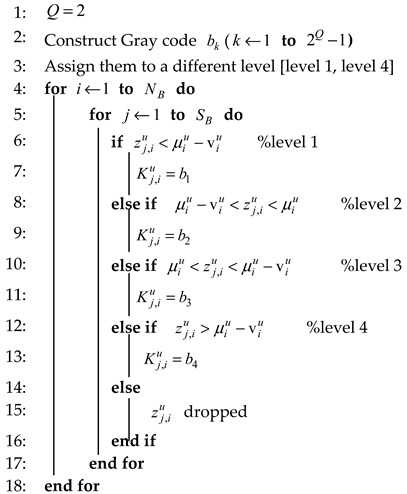



### 3.3. Performance Parameter

There are four parameters used to determine the performance of our proposed SKG scheme as follows:

1. Pearson correlation coefficient: calculates linear dependency between two RSS data. The resulting value ranges within −1 to 1, where −1 specifies a negative correlation, 0 specifies no correlation, while 1 specifies an absolute correlation [44]. In this paper, we use this parameter to determine the success of the MK method. This success is indicated by an increase in the correlation coefficient of legitimate users. Detailed testing is conducted by comparing the correlation coefficient of the RSS data block measurement results with the pre-process results by using the MK method. From the test results, there will be an increase in the correlation coefficient close to 1 from several data blocks. The closer to 1 the greater the probability of obtaining an identical secret key.
2. Bit disagreement rate (BDR): bit incompatibility of total bits in one RSS data block. This parameter is the first parameter used to determine the success of the CMQ method. In this paper, we make effort to build a simple SKG scheme by eliminating the information reconciliation stage. One of the requirements that must be fulfilled to eliminate this stage is that the obtained BDR must have a value of 0, where the value means the secret key produced is perfectly identical. Therefore, the SKG scheme does not require a correction process. Detailed testing is conducted by comparing the bits from multilevel quantization between two legitimate users. Quantization is carried out on each block of the pre-process RSS data.
3. Key generation rate (KGR): the number of produced bits at one time in the SKG scheme. This parameter is the second parameter used to determine the success of the CMQ method. KGR is obtained by calculating the number of secret key bits that have been successfully generated in a certain period of time. Thus, it can be said that the purpose of these parameters is to determine the speed of the built SKG scheme to acquire the secret key. In accordance with the 802.1x recommendation, the secret key must be refreshed every 1 h [45]. KGR value will meet the requirements if the secret key can be obtained within that time period.
4. Randomness: utilized to determine the randomness of the produced secret key. Testing of the *p* values of several parameters is conducted using the NIST statistical suite [46]. A secret key will pass the randomness requirements if p≥0.01. In this paper, we conducted 6 randomness tests out of a total of 15 tests that can be conducted using NIST. This test is selected because the secret key length only meets the requirements of the 6 tests. The remaining tests require very long bits. Even some tests need a bit length $\approx 10^{6}$. Because we performed randomness testing in each 128-bit secret key (used for AES-128), we merely had to undertake the testing with the 6 tests.

## 4. Experimental Setup

The SKG scheme is built on 3 Raspberry Pi 3 Type B devices. There are 2 devices that become legitimate users, i.e., Alice and Bob, while the other devices become eavesdroppers (Eve). Each device is equipped with TL-WN722N 802.11 b/g/n wireless card. The operating system used is Linux Raspbian Stretch with the kernel version 4.14.74. RSS data aggregation in the probing channel stage is conducted using Wireshark software. The amount of RSS data collected in both legitimate users and eavesdropper is 4000. In studies on the SKG scheme, Raspberry Pi is often used as a tool for processing RSS measurements so a shared secret key can be obtained [23,47]. The selection of this equipment is based on the ease of programming used because it uses high-level programming, i.e., Phyton, and expansion slots to facilitate connectivity. The ease of connectivity is needed in various IoT applications. Besides the SKG scheme, Raspberry Pi can also be used as part of a chaotic cryptosystem [17]. However, its use is different from the utilization of Raspberry Pi in the SKG scheme which processes all key generation processes. In this research, Raspberry Pi is only used as one of the subsystems that are connected to other devices, namely the digital camera, monitor, and Field-Programmable Gate Array (FPGA). FPGA is a digital IC that is often used to implement digital circuits. The selection of Raspberry Pi is based on the ability to develop graphical interfaces through python, so the mechanism of capture and display of images that will be encrypted/decrypted can be done.

### 4.1. Experimental Scenarios

**Figure 4:**
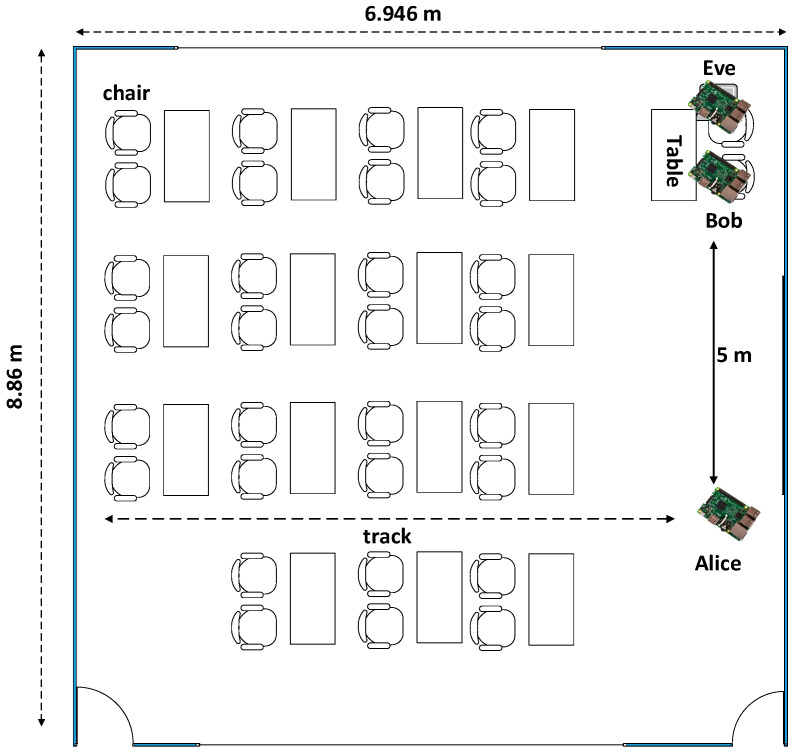
The experimental scenario in the line-of-sight (LOS) environment.

**Figure 5:**
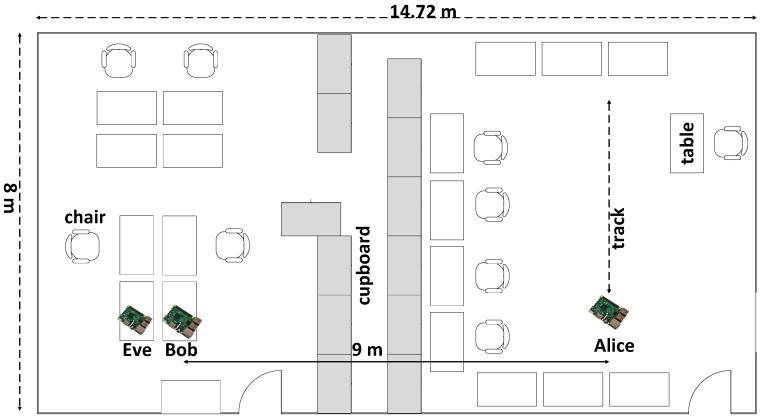
The experimental scenario in the non-line of sight (NLOS) environment.

There are two experiments to be performed at this paper, i.e., experiment 1 and 2. As seen in Figure 4, experiment 1 was carried out in the line-of-sight (LOS) environment, while Figure 5 shows that experiment 2 was carried out in a non-line-of-sight (NLOS) environment. In the LOS environment, testing was carried out in a room measuring 8.68 × 6.946 m with a table, chairs, and blackboard in it. Alice moved along the track, while Eve and Bob were motionless with a very close distance of 10 cm. There was no barrier between Alice and Bob. In the NLOS environment, testing was carried out in a sized room measuring 14.72 × 8 m with tables, chairs and glass cabinets inside. Alice moved along the track with a barrier in the form of a glass cabinet. Eve and Bob were also motionless with a distance of 10 cm. The distance had exceeded half the wavelength of 6.25 cm ($\lambda =c/f=3.10^{8}/2.4.10^{9}=12.5$ cm), so Eve would not get the same RSS data.

### 4.2. Measurement Results

The measurement results show a high correlation coefficient of the RSS data between legitimate users as seen in Table 1. In the LOS environment (Experiment 1) the correlation coefficient of 0.7573 was obtained. Meanwhile, in the NLOS environment (Experiment 2) the correlation coefficient of 0.6988 was obtained. The smaller correlation of RSS data in the NLOS environment indicates that the multipath component found in this environment causes an increase in the difference of the RSS data. The existence of a barrier in the form of cupboard between Alice and Bob causes the RSS data received to come from several paths (multipath), so the signal was weakened. These conditions led to the increasing differences in the RSS data of each user. The correlation coefficient obtained by eavesdroppers is very low (uncorrelated), so we can say that the built SKG scheme has fulfilled the spatial decorrelation principles, and it is difficult for an eavesdropper to obtain the same secret key as the legitimate user.

**Figure 6:**
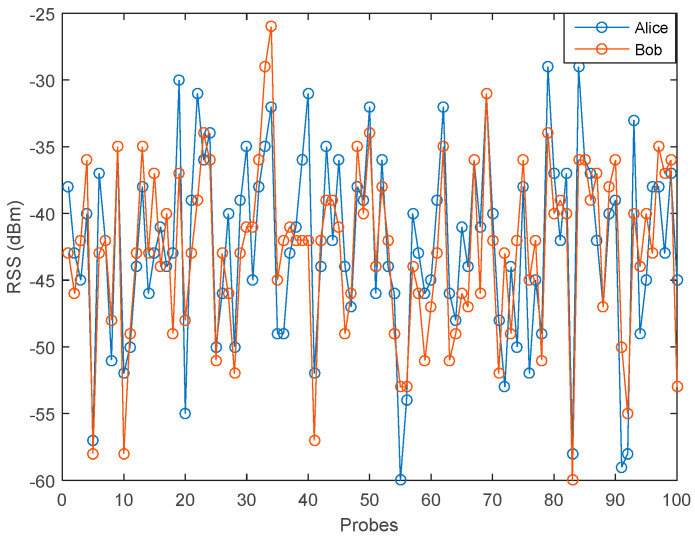
Measurement results in the LOS environment.

**Figure 7:**
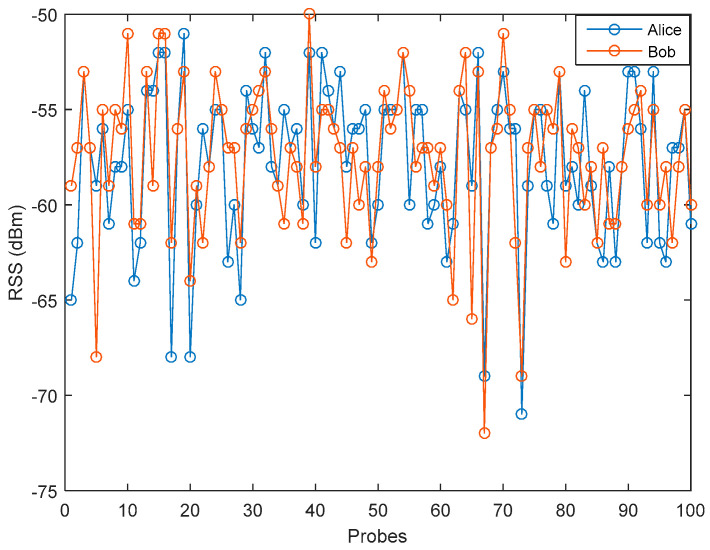
Measurement results in the NLOS environment.

Figure 6 and Figure 7 show variations of RSS data in the LOS and NLOS environment. The measurement results in the LOS environment tend to show a better strength signal variation compared to the NLOS environment which is between −25 dBm to −60 dBm. Meanwhile, the strength signal variation in the NLOS environment is between −50 to −75. This occurs because in the LOS environment there is no barrier between Alice and Bob, so the possibility of getting a stronger signal is greater than in the NLOS environment. Therefore, it can be said that the barrier and scatterer in the NLOS environment, cause the weakening of the received signal.

## 5. Performance Evaluation

In this segment, we assess the achievement of the built SKG scheme by using several predetermined parameters. This evaluation was used to determine the success of the proposed method, namely MK and CMQ. Experimental validation was carried out in two test environments, i.e., the LOS and NLOS environments. The success of the MK method is indicated by the increasing correlation coefficient of a legitimate user. Evaluation is conducted by comparing the correlation coefficient of the RSS data block of the measurement with the pre-process results using the MK method. The more blocks of data with a correlation coefficient of close to 1, the more likely it is to get an identical secret key. The success of the CMQ method is shown by the success of obtaining identical secret keys without requiring an information reconciliation stage. This stage can be omitted if there is a block of RSS data that has BDR with a value of 0 and the success of getting a secret key within the recommended time span of 802.1x [45]. The speed of the SKG scheme to get the secret key in that time period is indicated by the KGR parameter. In addition, we also carry out the NIST test to ensure that the generated secret key has *p* ≥ 0.01 to meet randomness requirements.

### 5.1. Performance Evaluation of the Modified Kalman (MK) Method

In this test, we divided the RSS data into several data blocks, i.e., 64 and 128. The purpose of this data block distribution is to ensure an improvement in the correlation of each data block so as to increase the similarity of the secret key produced. The selection of the amount of RSS data on each block is based on the quantization method used. We used a multilevel quantization method that converts 1 RSS data into 2 bits, so if 1 block contains 64 RSS data, then it will be converted to 128 bits. If 1 block contains 128 RSS data, then it will be converted to 256 bits. Because the length of the secret key used is 128 bits, the data block will be divided into 2 so each block contains 128 bits.

Table 2 demonstrates the achievement of the MK algorithm as opposed to the existing measurement results for all RSS data. The correlation coefficient obtained is the value of the entire RSS data after processing the RSS data for each block by using the MK method. The results of the experiment show that the RSS data distribution into several data blocks gives an increase in a correlation coefficient of the legitimate user in both LOS and NLOS environments. In the NLOS environment, blocks of data containing 64 RSS data resulted in a higher significant improvement in the correlation coefficient compared to the blocks of data containing 128 RSS data. However, there is a decrease in the correlation coefficient in the LOS environment. This condition occurs because of a decrease in the correlation coefficient when processing data using the adopted polynomial regression (Algorithm 1), so when the data were processed using the Modified Kalman Filter (Algorithm 2) there is no improvement in the correlation coefficient even tends to decline. In this paper, we select blocks of data containing 128 RSS data because of the improvement in the correlation coefficient in all environments. Eavesdropper’s correlation coefficients also increase, but the results obtained are still far below the correlation coefficient obtained by legitimate users. Therefore, it is still difficult for eavesdroppers to get an identical secret key as legitimate users.

**Figure 8:**
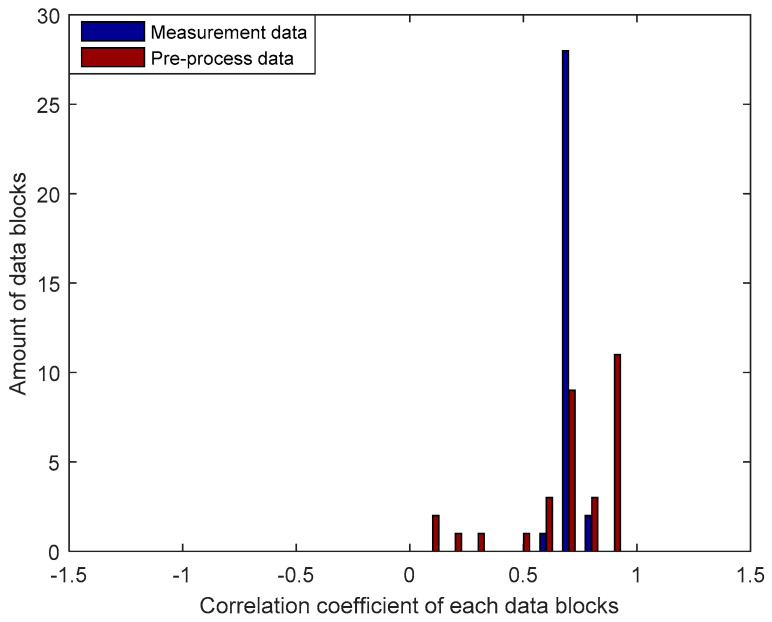
Improvement of the correlation coefficient for each block of data in the LOS environment.

We conducted a detailed analysis of the improvement in the correlation coefficient of the legitimate user with each block of data containing 128 RSS data as seen in Figure 8. Testing is conducted by comparing the correlation coefficients of each measured block of data with the pre-process results by using the MK method. The results of an experiment in the LOS environment indicate that most measurement data blocks have a correlation coefficient of 0.7. After the pre-process stage, there is a significant increase in the number of data blocks that have a correlation coefficient of 0.9 with a range value between 0.9016 to 0.9996. There are 2 blocks of data that have the correlation coefficients of 0.9993 and 0.9996, so it is possible to obtain an identical secret key from the two blocks of data without requiring the information reconciliation stage.

**Figure 9:**
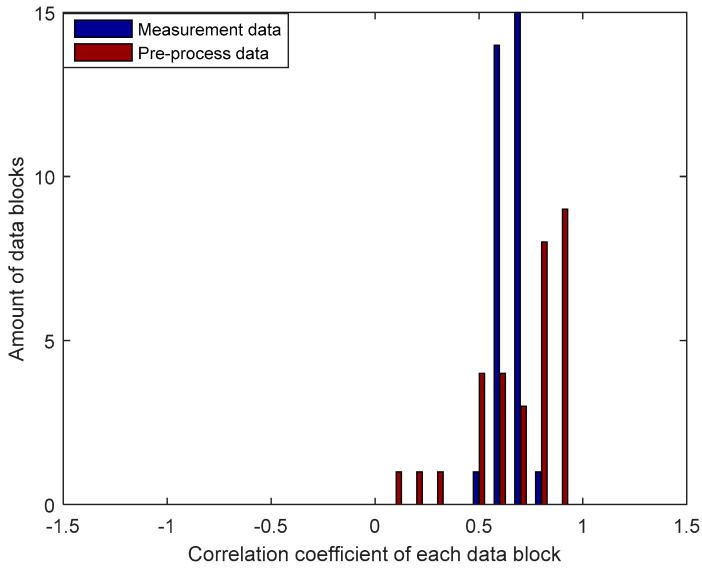
Improvement of the correlation coefficient for each block of data in the NLOS environment.

The test results of RSS data for each block in the NLOS environment as seen in Figure 9 also showed an improvement in the correlation coefficient of the pre-processes results when compared with the measured data. From the pre-process results, there are 9 blocks of data that have a correlation coefficient of 0.9 with a range of values between 0.9053 to 0.9976. One block has a correlation coefficient of 0.9976 so it is possible to obtain an identical secret key without requiring an information reconciliation stage. Generally, the MK method produces a better improvement in the correlation coefficient of the pre-process results in the LOS environment. This can be seen from the increasing number of data blocks that have a correlation coefficient of 0.9 when compared to the NLOS environment, so it has a greater probability of producing identical secret keys.

From the overall tests that have been conducted, it can be concluded that our proposed pre-process method, i.e., the MK method, is able to increase the significant correlation coefficient in some data blocks to 0.9. This increase has an effect on the greater the possibility of getting an identical secret key because of the increased similarity of the RSS data block resulting from the pre-processing stage. The increased number of data blocks with the correlation coefficient can reach up to 35.48% in the LOS environment and 29.03% in the NLOS environment. This shows the success of the addition of the MK method in the built SKG scheme since the method was able to increase the reciprocity of measured RSS data.

### 5.2. Performance Evaluation of the Combined Multilevel Quantization (CMQ) Method

In this segment, we oppose the achievement evaluation between our proposed method, i.e., CMQ and several existing methods/schemes. In our proposed method we utilize RSS data from the pre-process using the MK method $z^{u}$ as the input to be processed into the initial key $K^{u}$. The quantization method used is a multilevel quantization [48] that uses mean $\mu^{u}$ and variance $v^{u}$ to determine the level of each RSS data. There are 4 existing schemes used as a comparison, i.e., schemes [36,48,49,50]. Scheme [48] also uses mean and variance to determine the level of each RSS data, but the mean and variance are obtained from blocks of data containing 10 RSS data. The scheme uses RSS data from the pre-process using the existing Kalman as the input to be processed into the initial key. Scheme [49] uses intervals from sorted RSS data, where this scheme uses N=2 values as the number of bits to be extracted at each interval. Scheme [50] uses guard bands in each RSS data interval with values α as guard band comparison ratios with the total RSS data. We select α value of 0.1. The last existing scheme is [36], wherein this scheme is an enrichment of the scheme [48]. Compared to scheme [48] which uses 2 parameters, scheme [36] uses 3 parameters, namely the mean, standard deviation and α as a parameter that will be multiplied by the standard deviation. The α value used in this scheme is 0.01.

Performance evaluation of the SKG scheme is seen from several parameters, namely BDR, KGR, and randomness. BDR testing aims to determine bit incompatibility of total bits in one RSS data block. Since the built SKG scheme does not use the information reconciliation stage, the candidate secret key can be obtained if the BDR value is 0. KGR shows the number of bits produced at one time in the SKG scheme stage. The higher the KGR value is, the faster is the time needed to get the secret key. The randomness parameter aims to determine the level of randomness of the secret key generated. The level of randomness generated can be seen from the significance level α. The higher is the value α generated, the more random the secret key value is. In cryptographic systems, the minimum α value that must be fulfilled is 0.01 (p≥α).

**Figure 10:**
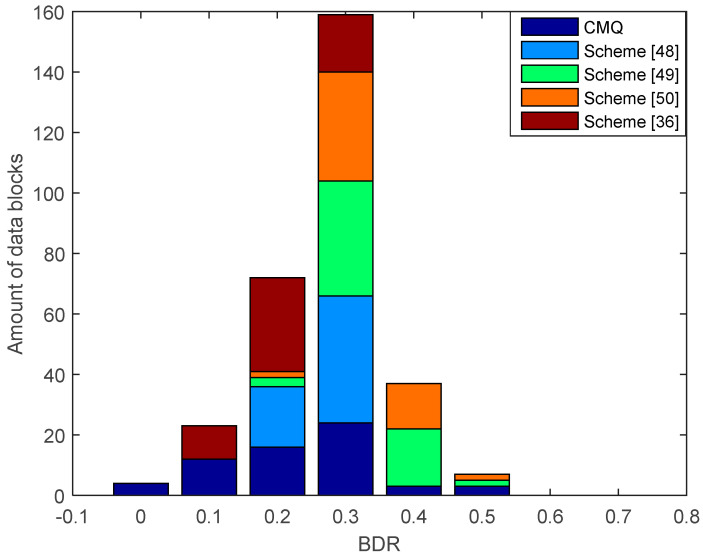
Bit disagreement rate (BDR) of the legitimate user between combined multilevel quantization (CMQ) and several existing schemes in the LOS environment.

**Figure 11:**
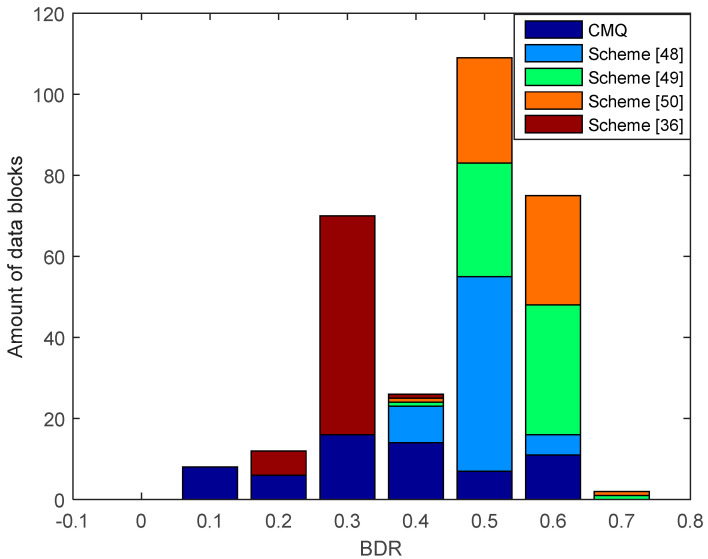
BDR of eavesdropper (Alice–Eve) between CMQ and several existing schemes in the LOS environment.

**Figure 12:**
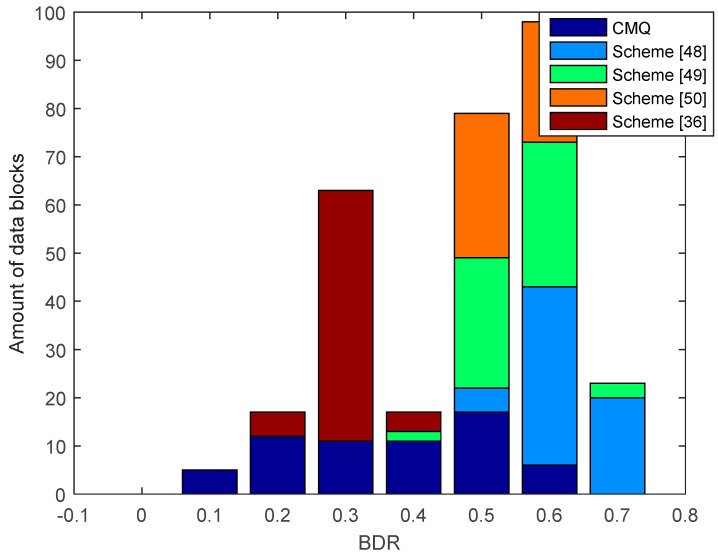
BDR of eavesdropper (Bob–Eve) between CMQ and several existing schemes in the LOS environment.

Figure 10 shows the results of the comparison of BDR between our proposed scheme, i.e., CMQ and several existing schemes in the LOS environment. BDR test results of the legitimate user indicate that our proposed scheme is capable of producing 4 identical candidate secret keys without requiring an information reconciliation stage since the BDR value is 0. This condition occurs because of an improvement in the correlation coefficient up to 0.9999 in several data blocks using the MK method. Increasing the correlation coefficient also increases the similarity of the pre-process RSS data results, thus improving the possibility to obtain an identical secret key without requiring an information reconciliation stage. The test outcomes also present that all of the existing schemes produce the non-identical secret key because all data blocks generate BDR values that exceed 0. Therefore, error correcting techniques are still needed to reconcile information. Figure 11 and Figure 12 show the BDR value between eavesdroppers and legitimate users. Many eavesdropper’s blocks of data have different bits with the legitimate user, so there is no BDR value that is worth 0. This shows that the eavesdropper does not get an identical secret key with the legitimate user.

**Figure 13:**
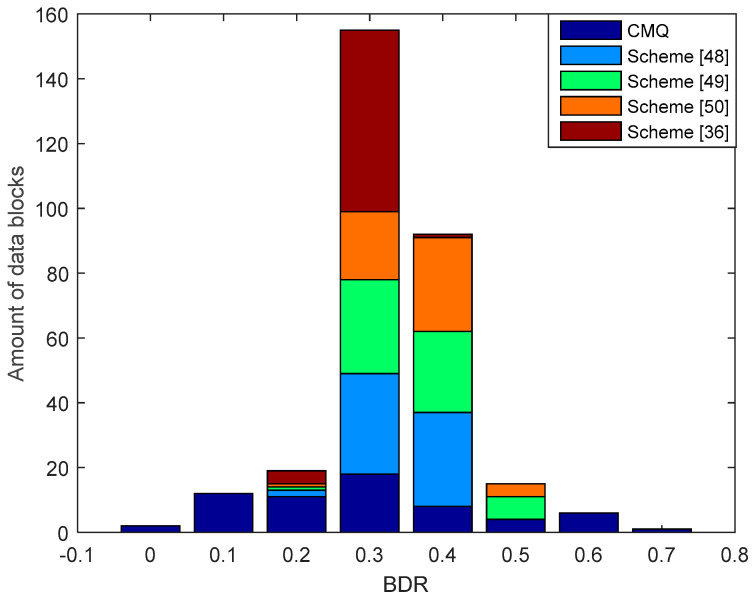
BDR of the legitimate user between CMQ and several existing schemes in the NLOS environment.

**Figure 14:**
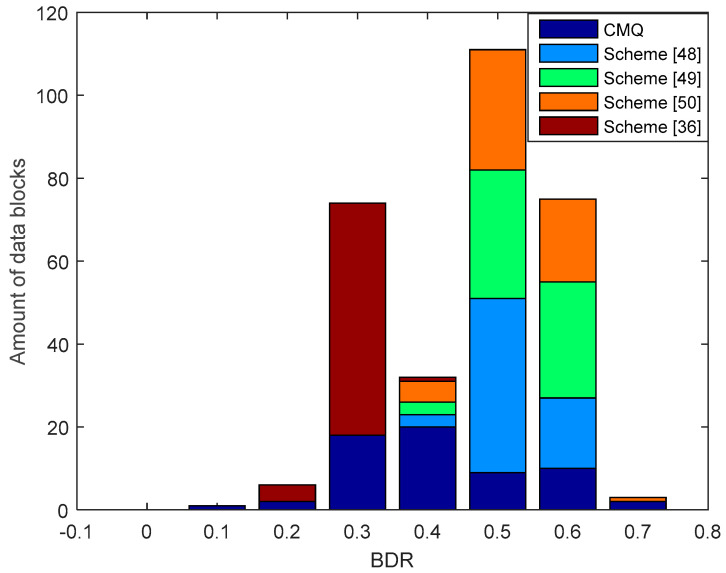
BDR of eavesdropper (Alice–Eve) between CMQ and several existing schemes in the NLOS environment.

**Figure 15:**
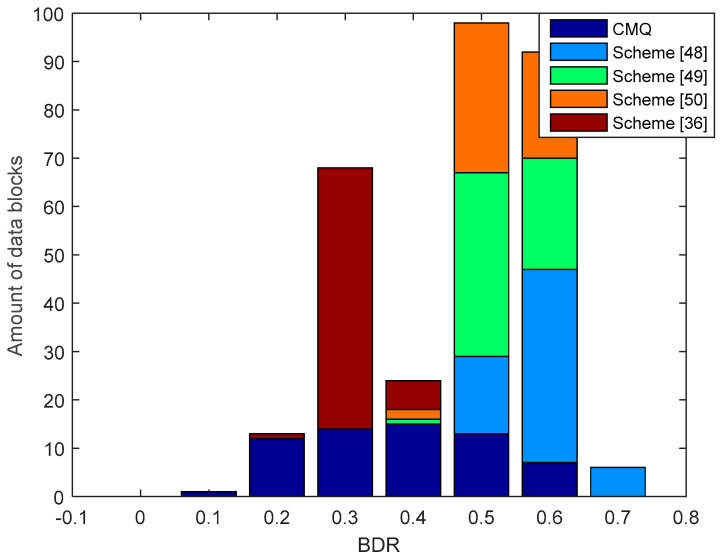
BDR of eavesdropper (Bob–Eve) between CMQ and several existing schemes in the NLOS environment.

Figure 13 presents the results of the comparison of BDR between our proposed scheme i.e., CMQ and several existing schemes in the LOS environment. BDR test results of the legitimate user indicate that our proposed scheme is capable of producing 2 identical candidate secret keys without requiring an information reconciliation stage because the BDR value is 0. The number of identical secret keys produced is still less when compared to testing in the LOS environment. This occurs because the overall correlation coefficient in the NLOS environment is still smaller when compared to the LOS environment. Besides, the number of data blocks that have increased the correlation coefficient up to 0.9 is also less when compared to the LOS environment. The test outcomes also show that all of the existing schemes produce the non-identical secret key because all data blocks generate BDR values that exceed 0. Therefore, error correcting techniques are still needed to reconcile the information. Overall, it can be appreciated that our scheme is able to produce a simpler SKG scheme compared to the existing scheme. This is indicated by the ability to obtain identical secret keys without going through the information reconciliation stage. Figure 14 and Figure 15 show BDR between legitimate users and eavesdroppers. The results of the tests indicate that there is no BDR that has a value of 0, so the eavesdropper does not get an identical secret key with the legitimate user. It shows that our proposed scheme is also able to warrant the security of the secret key generated by the legitimate user. The same with the testing in the LOS environment, the BDR values obtained by eavesdropper in the NLOS environment range between 0.5 and 0.7.

The next tested parameter is randomness by using the NIST statistical suite. There are 6 tests are carried out to ensure the randomness of a candidate secret key, which is generated from the privacy amplification stage. We provide a brief explanation of the objectives of each test as follows [46]. The approximate entropy test is used to determine the frequency of all possible overlapping bit patterns in a key sequence. The purpose of the frequency (monobit) test is to determine whether the proportions of 0 and 1 in a key sequence are the same. A frequency test within a block is used to determine whether the proportion of 1 in one block is around half a block. A run test is used to determine whether the oscillations of 1 and 0 of a key sequence are too fast or slow compared to a random sequence. A longest-run-of-ones in a block test determines whether the length of the 1 from the test sequence is consistent with the expected length of 1 from the random sequence. Cumulative sums test is used to determine whether the cumulative number of parts of the sequence is too large or too small for the expected cumulative number of a random sequence. Cumulative sums (forward) test use mode 0 by changing 0 to −1, while cumulative sums (reverse) test use mode 1 by changing 1 to +1.

From the test outcomes presented in Table 3, it can be ensured that all secret keys fulfill the randomness requirements with *p* value exceeding 0.01 for all types of tests. The priority of the selected key sequence as the shared secret key is key 4, key 3, key 1, and key 2. This selection is based on the approximate value of each key. If the first priority key failed in the verification stage, then the next key utilized as the secret key is the second priority, i.e., key 3. Generally, key 4 has a greater value for each type of test when compared to the other key. The approximate entropy test results show *p* values up to 0.980078. The higher the value of the test shows the higher the irregularity of the resulting bit so the resulting key sequence is more random. The highest *p* value of the frequency (monobit) test is 0.859684. The higher the test results show the proportions of 1 and 0 are almost the same or close to ½, so the distribution can be obtained in accordance with the requirements of randomness. On the frequency test within a block, the highest value is obtained on key 1 which is equal to 0.529508. This shows that key 1 has a proportion of 1, which is closer to half the block, so it is as expected on the randomness assumption. The results of the key 1 run test also show a greater *p* value compared to the other keys which are equal to 0.920091. These results indicate that the oscillations occurring in the key are faster when compared to other keys. The longest-run-of-ones in a block test shows that key 4 has a length of 1 that is more invariant with the expected length of 1 from a random key set. The results of cumulative sums testing (forward and backward) indicate that the cumulative number of produced keys corresponds to the expected cumulative number of a random sequence. Too many 1 or 0 at the beginning of the key sequence (mode 0) and at the end of the key sequence (mode 1) will result in the *p* value being too small, so it does not meet the randomness requirements.

The results of the NIST statistical suite randomization test in the NLOS environment are shown in Table 4. There are 2 keys with the first priority as a shared secret key, i.e., key 1 with an approximate entropy value of 0.916730. If the verification stage fails, key 2 can be used as an alternative key. Overall, it can be seen that the produced secret keys have fulfilled the randomness requirements because the *p* value has exceeded 0.01. Key 1 shows a higher irregularity compared to key 2. This is indicated by a higher *p* value of key 1 when compared to key 2 in the approximate entropy test. To fulfill randomization requirements, the key obtained must have a proportion of 1 and 0 that are close to ½. The results of testing frequency (monobit) indicate that key 1 has a proportion of 1 and 0 that are closer to ½, so it has a higher *p* value than key 2. The same results were also obtained in testing the frequency test within a block, where key 1 has a proportion of 1 which is closer to half the block so it has a higher value than key 2. In run testing it appears that key 2 oscillates faster than key 1, besides that key 2 also has a length of 1 which is more consistent with the expected length of 1 from a random key sequence. The same results were also obtained in the frequency test within a block, where key 1 has a proportion of 1 which is closer to half the block so it has a higher *p* value than key 2. In run testing it appears that key 2 oscillates faster than key 1, besides that key 2 also has a length of 1 which is more invariant with the expected length of 1 from the random key sequence. In cumulative sums testing it appears that key 2 has too many 1 or 0 at the beginning and at the end of the key sequence, so the resulting *p* value is smaller than key 1. Overall, the results of the NIST testing in the LOS and NLOS environment show *p* values that have exceeded 0.01. It means that the generated secret keys have meets the randomness requirements with confidence level reaching up to 99%.

KGR is a performance parameter that aims to determine the speed of the SKG scheme built to obtain the secret key. The KGR test results as shown in Table 5 showed a higher KGR result in the LOS environment, i.e., 0.92 bps so that it took approximately 2.32 min to get a 128-bit secret key that would be utilized to encrypt the message using the AES-128 method. In accordance with the recommendations of 802.1x, the secret key must be refreshed every 1 h so that the SKG scheme built has fulfilled the recommendation [45]. This is because the secret key generated is still less than 1 h, which is 2.32 min. The test results in the NLOS environment also still fulfill the requirements for the refresh key, because the time needed to obtain the 128-bit secret key is 4.74 min. The average of approximate entropy in both test environments ranges from 0.5 to 0.6, with a lower average of approximate entropy obtained in the NLOS environment. In the built SKG scheme, we eliminate the information reconciliation stage. The total computation time needed is 18.3 s (LOS) and 18.6 s (NLOS), while the information reconciliation stage using BCH (31.6) requires computing time up to 7.139 s (LOS) and 7.068 s (NLOS). We assume that the scheme was tested in good network conditions. The elimination of these stages can reduce computational time to 39.1% (LOS) and 38% (NLOS). If network conditions are poor, then the possibility of decreasing computational time is also greater than good network conditions because of the longer time needed to exchange parity bits.

From the overall test to determine the success of the CMQ method, it can be concluded that our proposed method is able to produce a simple SKG scheme by eliminating information reconciliation stage. This condition is indicated by the production of several blocks of data that have a BDR value of 0. The results of tests carried out in the LOS and NLOS environments also show that the time required to obtain the secret key is far below 1 h which is 2.32 min (LOS) and 4.74 min (NLOS) with KGR values reaching 0.92 bps (LOS) and 0.45 bps (NLOS). Therefore, it can be said that the built SKG scheme has been able to meet the recommendations of 802.1x because the secret key could be refreshed under 1 h. Reducing the stage of information reconciliation also affects the decrease in computational and communication cost.

## 6. Conclusions

In this paper, we offer a new pre-process method, i.e., modified Kalman (MK), and perform a combination of the method with a multilevel quantization, i.e., combined multilevel quantization (CMQ). We also carried out experimental validation in two test environments i.e., LOS and NLOS. The results of the tests conducted indicate that the addition of the MK method to the built SKG scheme was able to increase the number of data blocks that have a correlation coefficient above 0.9 to 35.48% in the LOS environment and 29.03% in the NLOS environment. Therefore, the probability of getting an identical secret key is also increasing. Furthermore, the CMQ method was able to simplify the stages of the built SKG scheme because the scheme was able to produce several identical secret keys without requiring an information reconciliation stage with KGR reaching 0.92 bps in the LOS environment and 0.45 bps in the NLOS environment. The results of the tests conducted also showed that reducing the information reconciliation stage could reduce computational and communication time to 39.1% in the LOS environment and 38% in the NLOS environment. In addition, the secret key produced has passed 6 randomness tests with *p* values exceeding 0.01 so it can fulfill the requirements for randomization of cryptosystems with confidence levels reaching up to 99%.

## Figures and Tables

**Table 1:** The correlation coefficient of the measurement result.

Experiment	User	Correlation coefficient
1	Alice and Bob	0.7573
	Alice and Eve	0.0216
	Bob and Eve	0.0153
2	Alice and Bob	0.6988
	Alice and Eve	0.0100
	Bob and Eve	0.0282

**Table 2:** Improvement of correlation coefficient by using the modified Kalman (MK) method.

Experiment	User	Measurement Correlation Coefficient	Improvement of the Correlation Coefficient for the Data Block	
			64	128
1	Alice and Bob	0.7573	0.7500	0.8196
	Alice and Eve	0.0216	−0.0062	0.2017
	Bob and Eve	0.0153	0.1657	0.1995
2	Alice and Bob	0.6988	0.7782	0.7667
	Alice and Eve	0.0100	0.3016	0.2922
	Bob and Eve	0.0282	0.3682	0.3876

**Table 3:** National Institute of Standards and Technology (NIST) test in the LOS environment.

NIST Test	*p* Value			
	Key 1	Key 2	Key 3	Key 4
Approximate Entropy	0.594945	0.012247	0.841149	0.980078
Frequency (Monobit)	0.288844	0.376759	0.723674	0.859684
Frequency Test within a Block	0.529508	0.079139	0.323897	0.418642
Runs	0.920091	0.188673	0.382288	0.721539
Longest-Run-of-Ones in a Block	0.390869	0.508286	0.495995	0.876990
Cumulative Sums (forward)	0.314554	0.737518	0.654761	0.892023
Cumulative Sums (backward)	0.431439	0.431439	0.949266	0.818770

**Table 4:** NIST test in the NLOS environment.

NIST Test	*p* Value	
	Key 1	Key 2
Approximate Entropy	0.916730	0.182499
Frequency (Monobit)	0.595883	0.021556
Frequency Test within a Block	0.756805	0.635908
Runs	0.463206	0.502046
Longest-Run-of-Ones in a Block	0.151703	0.202941
Cumulative Sums (forward)	0.892023	0.026657
Cumulative Sums (backward)	0.431439	0.034021

**Table 5:** Key generation rate (KGR) in the LOS and NLOS environment.

Experiment	KGR (bps)	Average of Approximate Entropy
1	0.92	0.607105
2	0.45	0.5496145
